# An ultra-endurance event leads to changes in circulating regulatory T-cells, CD4+ naïve and CD8+ effector memory T-cells in the 48 h post-race recovery period

**DOI:** 10.1007/s00421-024-05677-y

**Published:** 2024-11-27

**Authors:** Hannah Lithgow, Laura Gibson, Russell Wilson, Neil Guthrie, Lesley Ingram-Sills, Tom Clifford, Mark Ross

**Affiliations:** 1 School of Energy, Geoscience, Infrastructure and Society, Institute of Life and Earth Sciences, Heriot-Watt University, JMF7, Edinburgh, EH14 4AS UK; 2 School of Applied Sciences, Edinburgh Napier University, Edinburgh, UK; 3 Institute of Cellular Medicine, Newcastle University, Newcastle, UK; 4 School of Sport, Exercise, and Health Sciences, Loughborough University, Loughborough, UK

**Keywords:** Endurance, Lymphocytes, Exercise, Adaptive immunity, T-cells

## Abstract

**Purpose:**

Exercise is known to acutely affect T-lymphocyte populations in the peripheral blood, which is intensity- and duration-dependent. However, effects of longer duration endurance exercise (>5 h) on T-cells in the days following are unknown. The aim of this study was to investigate the circulating T-cell changes that occur in response to an ultra-endurance event, which may provide insight into the inflammatory response to ultra-endurance exercise.

**Methods:**

Ten individuals (m = 7, f = 3) completing an Ironman 70.3 event volunteered for the study. Peripheral blood samples were taken 1–2 days pre-race (PRE-RACE), and 1 day (RACE + 1) and 2 days (RACE + 2) post-race, with circulating T-cells enumerated by flow cytometry (total CD3+, CD4+ and CD8+ T-cells, regulatory T-cells [CD4+CD25+CD127−; T_REG_], naïve [CD27+CD45RA+; NA], central memory [CD27+CD45RA−; CM], effector memory [CD27−CD45RA−; EM], and effector memory CD45RA+ [CD27−CD45RA+; EMRA]).

**Results:**

There were no changes in total CD3+, CD4+ and CD8+ T-cells. T_REG_ RACE + 1 was significantly higher compared to PRE-RACE, as were the proportion of CD4+ NA cells and CD8+ CM cells at RACE + 2; CD8+ EM cells fell at RACE + 2 (absolute counts and proportion).

**Conclusion:**

In conclusion, the ultra-endurance event evoked T-cell changes over the 48 h recovery period, with an increase in T-cells that regulate the immune response, and a reduction in circulating EM T-cells, most likely trafficked to sites of tissue damage and inflammation.

## Introduction

It is well established that long duration (>1.5 h) strenuous exercise can modulate immune function (Nieman 2007). Such bouts of exercise may affect immune function via modulating neutrophils (Quindry et al. 2003), monocyte/macrophage (Slusher et al. 2018) and/or lymphocyte function (Shaw et al. 2018). Indeed, we consistently observe drastic changes in T-cell populations in response to acute exercise bouts, with a dramatic rise in circulating T-cells immediately post-exercise (lymphocytosis), returning to baseline, or even below baseline (lymphocytopenia) within 30–60 min after the cessation of exercise (Ross et al. 2016; Turner et al. 2010). These effects are largely due to exercise-induced catecholamine release, for example, increased β_2_ adrenergic signalling (Dimitrov et al. 2010; Kruger et al. 2008) as a result of elevated circulating epinephrine and norepinephrine (Anane et al. 2009).

It is unlikely that the lymphocytopenia observed in the 30–60 min post-exercise period is reflective of depressed immune function (Campbell and Turner 2018, 2019), as cells are most likely redistributed to lymph tissues, lung and gut for immune surveillance (Kruger and Mooren 2007) or skeletal muscle to help coordinate muscle repair (Deyhle and Hyldahl 2018). We observed increased circulating T-cell subsets (namely CD4+ T-helper cells and regulatory T-cells [T_REG_]) 24 h post-marathon (Clifford et al. 2017), potentially indicative of greater immune surveillance, and regulation of the immune response to tissue damage and inflammation. However, due to the T-cell pool consisting of a wide variety of subsets, and the fact that these subsets respond differently to exercise (Simpson et al. 2007), it is likely that a long-duration, endurance exercise bout stimulates divergent responses across T-cell phenotypes. Therefore, the aim of the current study was to investigate the influence of a strenuous, long-duration endurance event (Ironman 70.3 race) on a wide range of circulating T-cell subsets (including total CD3+, CD4+, CD8+ T-cells, T_REG_, and naïve [NA], central memory [CM], effector memory [EM], and effector memory CD45RA+ [EMRA] cells). It was hypothesised that the ultra-endurance event would lead to significant elevations in cytotoxic and effector T-cells in the 48 h post-event.

## Materials and methods

### Ethical approval

The authors confirm that the study was performed in accordance with the ethical standards as laid down in the 1964 Declaration of Helsinki and its later amendments. Ethical approval was granted by the Edinburgh Napier University Research and Ethics Governance Committee. Written informed consent was obtained from all participants prior to commencement of the study.

### Participants

Ten (m = 7, f = 3) participants, aged 22–48 years, non-obese (<28 kg m^2^), normotensive (blood pressure < 140/90 mmHg) volunteered to take part in the study. All participants were already enrolled in an Ironman 70.3 (IM70.3) event prior to volunteering for the study. Participants visited the Human Performance Laboratory 1–2 days prior to the race for blood sampling (PRE-RACE), as well as the following 2 mornings after the race (RACE + 1, RACE + 2, respectively). Participants visited the lab between 7:30 a.m. and 9:00 a.m. on each day in a fasted state for peripheral blood sampling and other laboratory measures. Baseline characteristics are shown in Table 1.

**Table 1:** Participant characteristics and exercise trial data

	Participants (*n* = 10, 7 = m, 3 = f)
Age (years)	40 ± 9
Body mass index (BMI; kg m^2^)	22.2 ± 2.0
Systolic blood pressure (mmHg)	120 ± 7
Diastolic blood pressure (mmHg)	71 ± 2
$\dot{V}$O_2_peak (mL kg min^−1^)	56.5 ± 5.3
Power output @ $\dot{V}$O_2_peak (W)	347 ± 44
Power output @ 4 mmol L^−1^ BLa (W)	244 ± 46
$\dot{V}$O_2_ @ 4 mmol L^−1^ BLa (% of $\dot{V}$O_2_peak)	78.7 ± 8.4
Race time (hh:mm:ss) [range]	5:53:44 [05:30:23–6:20:28]

Values shown are mean ± standard deviation

*BLa* blood lactate

### Assessment of peak oxygen consumption and lactate threshold

Within 3 weeks of the race, but no closer than 1 week of the race, participants underwent an incremental cycling exercise test on a magnetically braked cycle ergometer (Velotron, RacerMate, USA) to volitional exhaustion to quantify lactate threshold and maximum oxygen consumption ($\dot{V}$O_2_max). The test began at 100 W for males, and 75 W for females, and increased by 25 W every 3 min to quantify lactate threshold (Messias et al. 2018), and conducted in line with recommendations from Bentley et al. (2007). Blood lactate (BLa) was measured using capillary finger prick blood samples using a portable lactate analyser (Lactate Pro 2; Arkray Inc., Japan) at the end of each stage. Once the participant reached or surpassed BLa of 4 mmol L^−1^, the intensity of exercise was increased by 25 W every minute to exhaustion. The intensity (%$\dot{V}$O_2_max) at which the participant exhibited a BLa of 4 mmol L^−1^ was recorded. Heart rate (HR) was monitored using HR telemetry (Polar, Finland).

### Blood sampling and T-cell phenotyping

Fasting blood samples were taken from participants 1–2 days prior to the race (PRE-RACE), and the two mornings after the race (RACE + 1, RACE + 2) by a trained phlebotomist using venepuncture. Peripheral blood was drawn into 6 mL vacutainers spray coated with EDTA anti-coagulant (BD Biosciences, UK), with the first 3 mL of peripheral blood discarded. Total blood differential leukocyte counts were determined using an automated haematology analyser (XS 1000i, Sysmex, UK). Peripheral blood mononuclear cells (PBMC) were isolated using density gradient centrifugation as described elsewhere (Ross et al. 2016). To quantify T_REG_ cells, cells were analysed on the day of blood collection. For the remainder of T-cell subsets, cells were frozen in RPMI and 10% dimethyl sulfoxide at −80 °C until batch analysis.

**Fig. 1:**
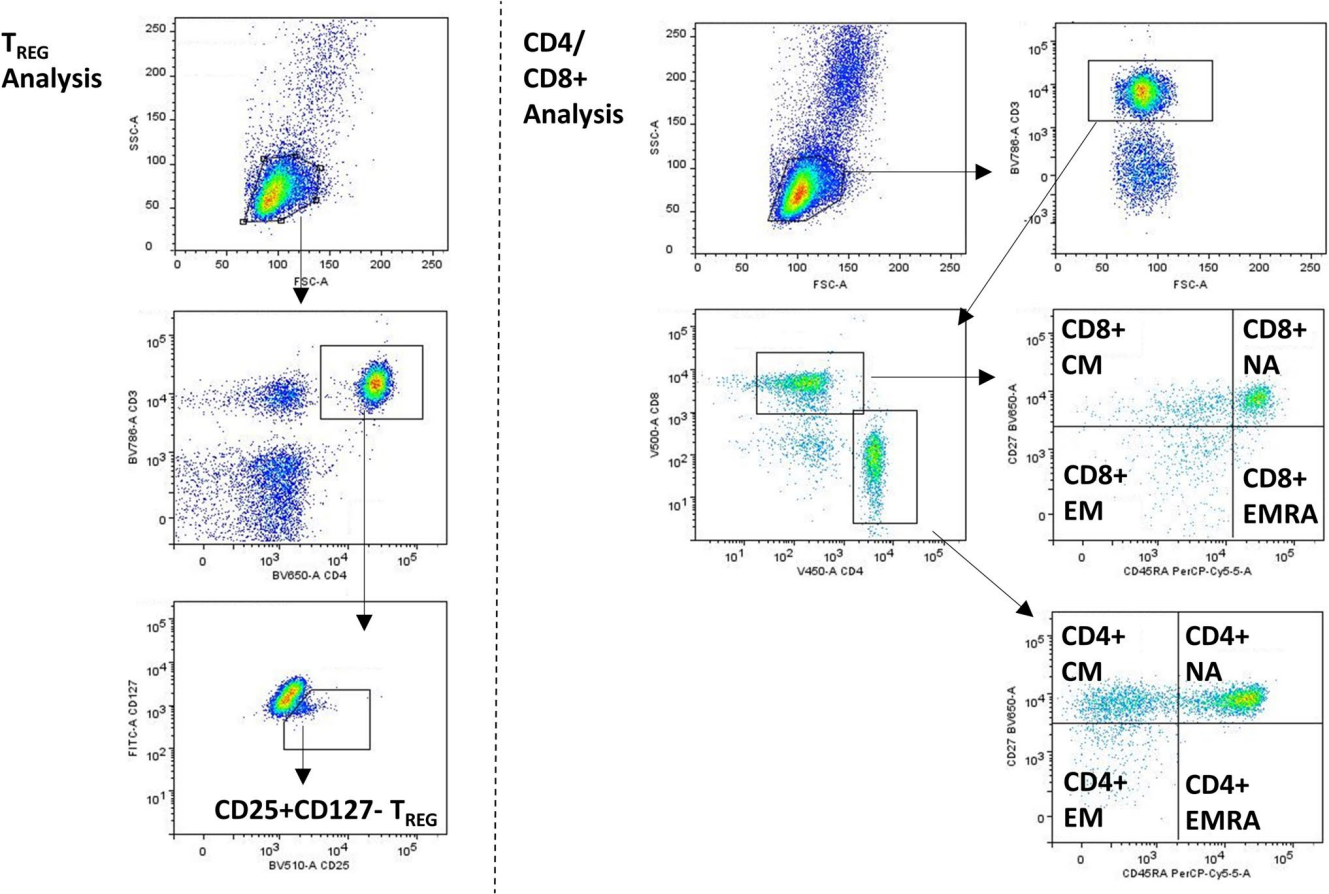
Flow cytometric quantification of T-lymphocyte populations. Side scatter vs. forward scatter for identification of lymphocyte gate, followed by gating CD3+ events. Subsequent gating for CD4+ T_REG_ are shown (CD25+CD127−), and CD4+ and CD8+ NA (CD27+CD45RA +), CM (CD27+CD45RA−), EM (CD27−CD45RA−), and EMRA (CD27−CD45RA +) events are shown

For T_REG_ cell analysis, PBMCs were stained with monoclonal antibodies anti-CD3, anti-CD4-BV650, anti-CD25-BV510, and anti-CD127-FITC (all BD Biosciences, UK) and left to incubate at 4 °C in the dark for 30 min prior to enumeration by flow cytometry (BD FACS Celesta, BD Biosciences, UK). T_REG_ cells were defined as CD3+CD4+CD25+CD127− cells (see Fig. 1 for flow cytometry gating strategy).

For CD4+ and CD8+ NA, CM, EM and EMRA phenotyping, these were performed in batch analysis on stored PBMCs. Frozen PBMCs were thawed on ice and subsequently stained with monoclonal antibodies against anti-CD3 BV786, anti-CD4 V450, anti-CD8 V500, anti-CD27 BV650 and anti-CD45RA PerCP Cy5.5 (all BD Biosciences, UK). NA, CM, EM and EMRA cells were defined as follows: CD27+CD45RA+, CD27+CD45RA−, CD27−CD45RA−, CD27−CD45RA+, respectively (Table 2). Cells were incubated with antibodies for 30 min at 4 °C prior to enumeration by flow cytometry. A minimum of 100,000 mononuclear cells were enumerated per sample for each T-cell panel. Flow cytometric gating strategy is shown in Fig. 1.

**Table 2:** T-cell population phenotyping

T-cell population	Phenotype
Total T-cells	CD3+
CD4+ T-cells	CD3+CD4+
CD8+ T-cells	CD3+CD8+
T_REG_	CD3+CD4+CD25+CD127−
CD4+ NA	CD3+CD4+CD27+CD45RA+
CD4+ CM	CD3+CD4+CD27+CD45RA−
CD4+ EM	CD3+CD4+CD27−CD45RA−
CD4+ EMRA	CD3+CD4+CD27−CD45RA+
CD8+ NA	CD3+CD8+CD27+CD45RA+
CD8+ CM	CD3+CD8+CD27+CD45RA−
CD8+ EM	CD3+CD8+CD27−CD45RA−
CD8+ EMRA	CD3+CD8+CD27−CD45RA+

*T*_*REG*_ regulatory T-cells, *CM* central memory, *EM* effector memory

### Statistical analysis

All data were assessed for normality using the Shapiro–Wilk test for normality. All data were deemed to be normal for subsequent analyses. For comparisons between PRE-RACE, RACE + 1, and RACE + 2 for all cell populations, several one-way repeated measures analyses of variance (ANOVA) were performed. Main effects of time were determined, and where there were significant main effects and where there were significant main effects, Tukey’s multiple comparisons tests were performed to detect specific differences across the different visits (PRE-RACE, RACE + 1, RACE + 2). Data was analysed using SPSS Statistics for Windows (SPSS v26, IBM, Corp, New York, USA) and figures designed using GraphPad (GraphPad Prism 6.4.1, Dotmatics, USA). Significance alpha was set at *p* < 0.05. All data are presented as mean ± SD unless otherwise stated.

## Results

### Influence of IM70.3 race on peripheral blood mononuclear cells

Our data show that there were significant elevations in circulating neutrophils and monocytes 1 day post-race compared to pre-race (neutrophils: PRE-RACE 2200 ± 777 cells μL^−1^ vs. RACE + 1 3249 ± 875 cells μL^−1^, *p* = 0.001; monocytes: PRE-RACE 422 ± 145 cells μL^−1^ vs. RACE + 1 585 ± 174 cells μL^−1^, *p* = 0.002). Both neutrophils and monocytes returned to similar to baseline levels after 48 h post-race. Total lymphocyte numbers did not change across the 3 days (see Table 3).

**Table 3:** Changes in circulating leukocyte number in response to ultra-endurance event

	PRE-RACE	RACE + 1	RACE + 2	Main effects (*F* value, *p* value)
Neutrophils	2200 ± 777	3249 ± 875^δ,γ^	2399 ± 707	10.590, 0.001**
Monocytes	422 ± 145	585 ± 174^δ,γ^	466 ± 123	9.007, 0.002**
Lymphocytes	1668 ± 363	1836 ± 506	1684 ± 478	2.190, 0.141

Values shown are mean ± SD

** *p* < 0.001 main effect

^δ^Significantly different from PRE-RACE

^γ^Significantly different from RACE + 2

### Influence of IM70.3 race on T-lymphocyte subpopulations

There was no effect of the ultra-endurance event on absolute counts (cells μL^−1^) of peripheral blood CD3+ T-cell (*F* = 2.582, *p* = 0.103), CD4+ T-cells (*F* = 3.266, *p* = 0.062), or CD8+ T-cells (*F* = 0.209, *p* = 0.814). There were no significant changes in proportion of CD4+ cells (% of CD3+) (*F* = 2.191, *p* = 0.141), however, there was an increase in proportion of CD8+ T-cells (% of CD3) from RACE + 1 to RACE + 2 (main effect *F* = 5.462, *p* = 0.014, RACE + 1: 28.1 ± 6.7%, RACE + 2: 31.9 ± 8.1%, *p* = 0.011). Data are shown in Table 4.

**Table 4:** Changes in circulating T-cell populations in response to ultra-endurance event

	PRE-RACE	RACE + 1	RACE + 2	Main effect (*F* value, *p* value)
CD3+ T-cells				
Cells μL^−1^	1184 ± 310	1325 ± 463	1160 ± 460	2.582, 0.103
CD4+ T-cells				
Cells μL^−1^	731 ± 195	851 ± 299	707 ± 273	3.266, 0.062
% of CD3+	63 ± 11	65 ± 9	62 ± 10	2.191, 0.141
CD8+ T-cells				
Cells μL^−1^	365 ± 154	381 ± 174	372 ± 174	0.209, 0.814
% of CD3+	30 ± 9	28 ± 7^γ^	32 ± 8	5.462, 0.014*

Values shown are mean ± SD

*p < 0.005, main effect

^γ^Significantly different from RACE + 2

There were no significant changes in absolute counts of CD4+ NA (*F* = 1.041, *p* = 0.373), CD4+ CM (*F* = 2.626, *p* = 0.100), CD4+ EM (*F* = 3.414, *p* = 0.055), or CD4+ EMRA cells (*F* = 1.735, *p* = 0.205). Likewise, there were no significant changes in absolute counts of CD8+ NA (*F* = 1.013, *p* = 0.383), CD8+ CM (*F* = 3.375, *p* = 0.057), or CD8+ EMRA cells (*F* = 1.459, *p* = 0.259). There was a significant decline in absolute counts of CD8+ EM cells from PRE-RACE to RACE + 2 (main effect *F* = 3.929, *p* = 0.038; PRE-RACE: 110 ± 77 cells μL^−1^, RACE + 2: 75 ± 29 cells μL^−1^, *p* = 0.040).

There were largely no significant changes in proportional data (cells as % of parent cell, e.g. % of CD4+ or % of CD8+). There were no changes in CD4+ CM (*F* = 0.229, *p* = 0.799), EM (*F* = 1.444, *p* = 0.262), EMRA (*F* = 1.29, *p* = 0.299), or CD8+ NA (*F* = 0.733, *p* = 0.494), or EMRA (*F* = 2.902, *p* = 0.081) cells, but noted increases in proportion of CD4+ NA and CD8+ CM from RACE + 1 to RACE + 2 (CD4+ NA: main effect *F* = 3.978, *p* = 0.037; RACE + 1: 52.0 ± 9.6%, RACE + 2: 57.0 ± 11.8%, *p* = 0.041; CD8+ CM: main effect *F* = 5.453, *p* = 0.014; RACE + 1: 13.0 ± 10.8%, RACE + 2: 20.7 ± 8.4%, *p* = 0.016), with a significant drop in proportion of CD8+ EM cells from PRE-RACE to RACE + 2 (main effect *F* = 4.041, *p* = 0.036; PRE-RACE: 28.8 ± 11.0%, RACE + 2: 21.9 ± 6.9%, *p* = 0.046).

**Fig. 2:**
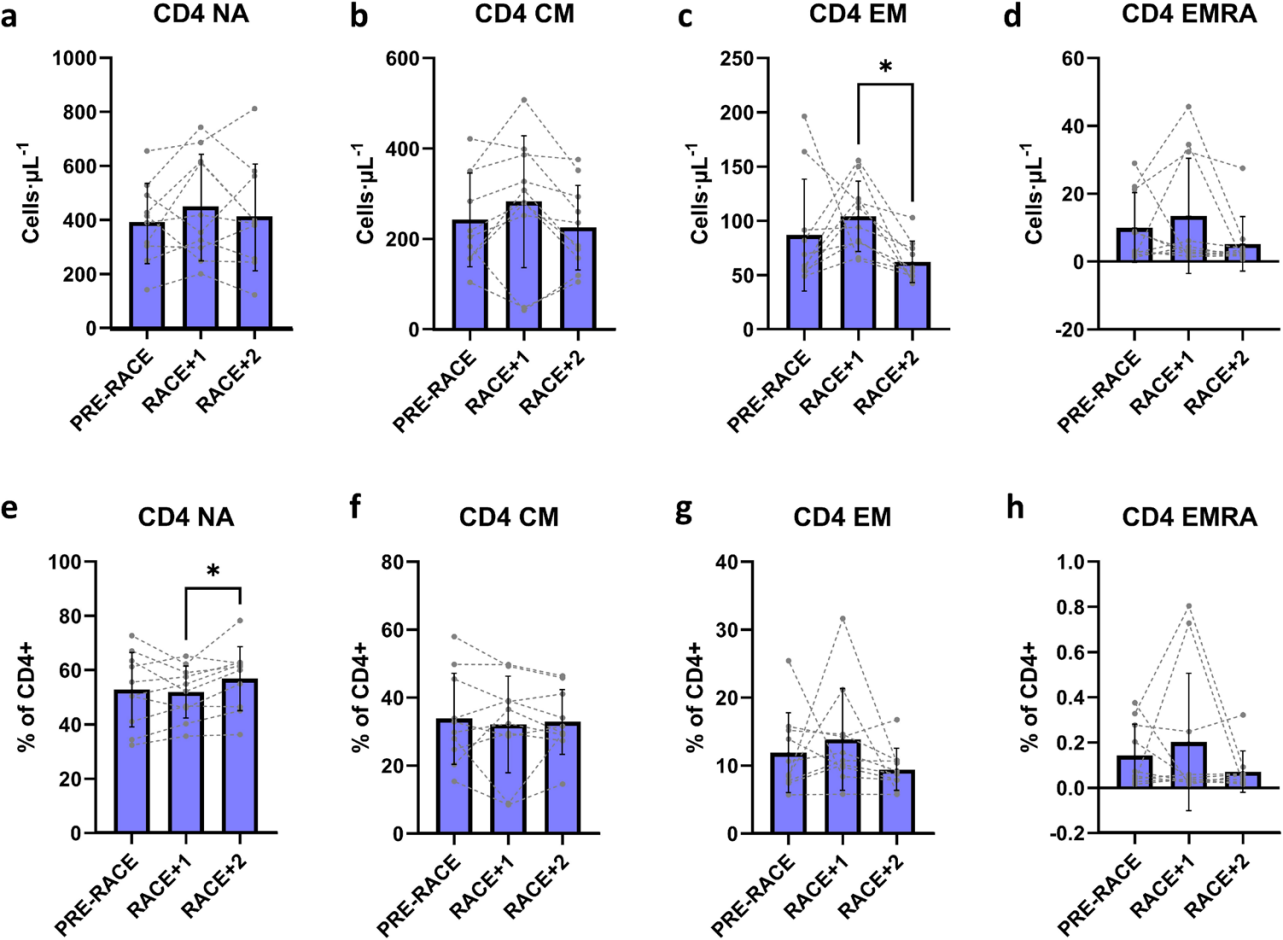
CD4+ T-cell changes in 48-h post-exercise period in response to ultra-endurance race (*n* = 10). CD4+ naïve (CD4+ NA, **a**), central memory (CD4+ CM, **b**), effector memory (CD4+ EM, **c**) and effector memory CD45RA+ (CD4+ EMRA, **d**) absolute counts over 3 days (PRE-RACE, RACE + 1, RACE + 2). Corresponding proportional data are shown in **e**–**h**. Values shown are mean ± SD and individual datapoints, * *p* < 0.05

**Fig. 3:**
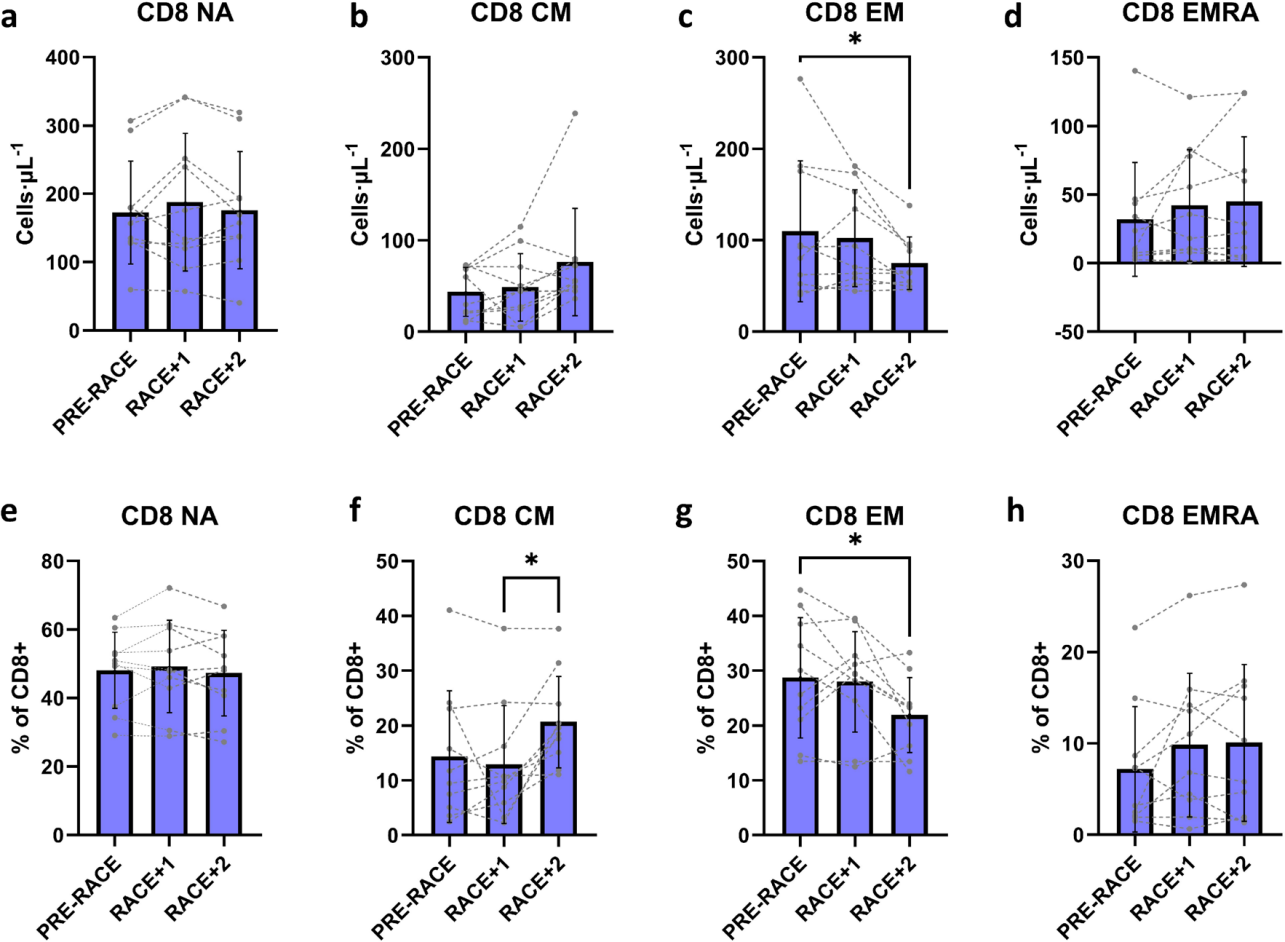
CD8+ T-cell changes in 48-h post-exercise period in response to ultra-endurance race (*n* = 10). CD8+ naïve (CD8+ NA, **a**), central memory (CD8+ CM, **b**), effector memory (CD8+ EM, **c**) and effector memory CD45RA+ (CD8+ EMRA, **d**) absolute counts over 3 days (PRE-RACE, RACE + 1, RACE + 2). Corresponding proportional data are shown in **e**–**h**. Values shown are mean ± SD and individual datapoints, * *p* < 0.05

CD4+ and CD8+ T-cell data are shown in Figs. 2 and 3.

**Fig. 4:**
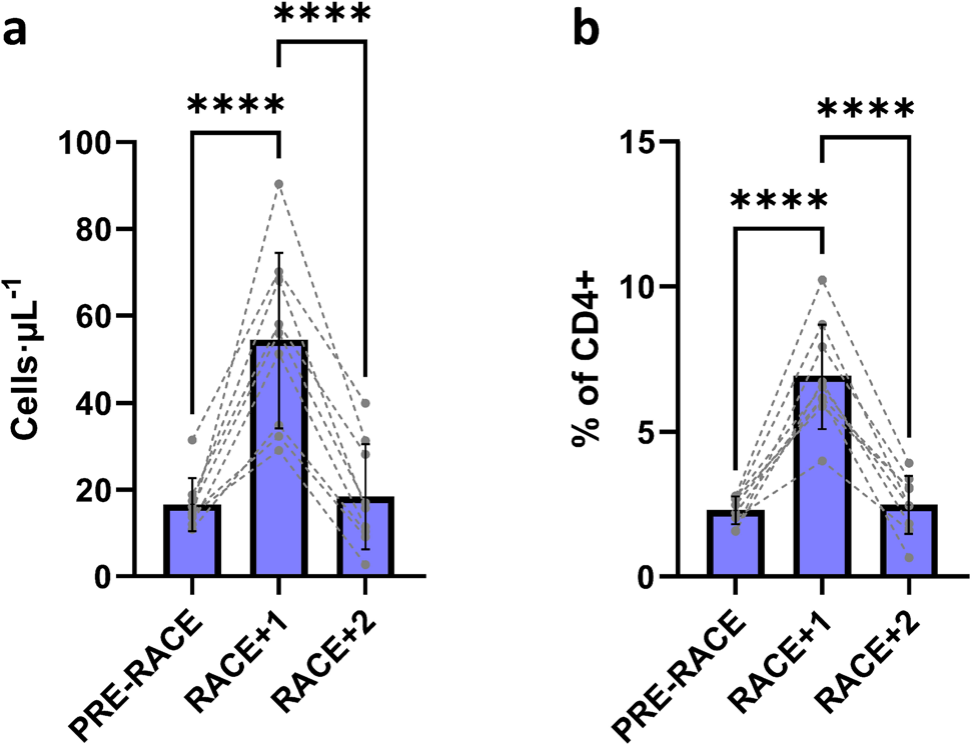
CD4+ regulatory T-cell (CD4+ T_REG_) changes in 48-h post-exercise period in response to ultra-endurance race (*n* = 10). **a** absolute counts over 3 days (PRE-RACE, RACE + 1, RACE + 2), **b** corresponding proportional data. Values shown are mean ± SD and individual datapoints, **** *p* < 0.001

Despite no changes in total CD4+ cell number, circulating T_REG_ cells were significantly elevated on RACE + 1 compared to PRE-RACE (absolute counts: main effect *F* = 41.730, *p* < 0.001; PRE-RACE: 16 ± 6 cells μL^−1^, RACE + 1: 55 ± 20 cells μL^−1^, *p* < 0.001; proportional data as % of CD4+: main effect *F* = 61.230, *p* < 0.001; PRE-RACE: 2.3 ± 0.5%, RACE + 1: 6.9 ± 1.8%, *p* < 0.001). These values returned to baseline levels at RACE + 2. T_REG_ data are shown in Fig. 4.

## Discussion

Our data show that an ultra-endurance event (IM 70.3), significantly altered circulating leukocytes in the 2 days after the event. Namely, there were elevations in neutrophils and monocytes (RACE + 1 vs. PRE-RACE), possibly reflective of inflammatory response to extreme exercise (Comassi et al. 2015; Shin and Lee 2013; Stelzer et al. 2015), but also alterations in specific T-lymphocyte subsets, with elevations in T_REG_, CD4+ NA, CD8+ CM, and a drop in CD8+ EM cells, with no other alterations in other T-lymphocyte subsets (CD4+ CM, EM, EMRA, CD8+ NA, EMRA).

The neutrophil and monocyte data suggest a strong inflammatory response to the ultra-endurance bout. Acute exercise is known to increase neutrophils and monocytes, which can be elevated for up to 6–24 h post-exercise (Peake et al. 2017; Walsh et al. 2011). In this study both neutrophils and monocytes were elevated at 24 h post-race (RACE + 1), which returned to near baseline (PRE-RACE) levels by 48 h post-race (RACE + 2). The neutrophilia may be due to cortisol-stimulated bone marrow release (McCarthy and Dale 1988), or other inflammatory factors which are also responsible for mobilisation of cells from the bone marrow, such as interleukin-6 (IL-6), glucocorticoids, and granulocyte colony stimulating factor (Suzuki et al. 2003). These neutrophils, once in the circulation, can be attracted to muscle damage by chemoattractants (Tsivitse et al. 2005), and subsequently the cells migrate into the affected muscle tissue (McLoughlin et al. 2003). Monocyte elevations are also likely due to increased bone marrow production and release (Shi and Pamer 2011), which also infiltrate skeletal muscle after tissue damaging exercise (Marklund et al. 2013; McLoughlin et al. 2003) such as ultra-endurance bouts (Marklund et al. 2013), subsequently transitioning into macrophages. These tissue infiltrating macrophages contribute to tissue repair and regeneration, and without this process of immune cell infiltration, recovery from tissue damaging exercise is limited (Tidball and Wehling-Henricks 2007). Studies have demonstrated that monocytes/macrophages can contribute to tissue repair via clearing debris (Arnold et al. 2007), stimulating muscle satellite cell differentiation (Tidball and Wehling-Henricks 2007), and promoting angiogenesis (Latroche et al. 2017; Ochoa et al. 2007)thus, these cells are a key player in the recovery from ultra-endurance exercise, where tissue damage is extensive (Rubio-Arias et al. 2019).

This is the first study to enumerate specific circulating T-cell subsets in the days after an ultra-endurance event. Previous work has demonstrated that exercise results in an acute lymphocytosis during exercise followed by lymphocytopenia in the minutes post-exercise (Rooney et al. 2018), which is likely to have occurred in this study. The longer-term changes (days post-race vs. minutes/hours post-race) are likely reflecting the chronic inflammatory processes taking place in muscle, lung and/or other peripheral tissues that result from such exercise. Turner et al. (2013) observed elevated C-reactive protein (CRP) after a single-stage, multi-day 233 km running event (100-fold for 24 h, eightfold after 7 days post-race), and Rubio-Arias et al. (2019), whilst also observing elevations in CRP over 72 h post-ultra race, observed significant muscle damage (plasma creatine kinase) over the same timepoints. These studies and ours indicate that an ultra-endurance event represents a significant inflammatory stimulus, which could be contributing to the peripheral blood immune cell components, due possibly to trafficking of key immune cell subsets into inflamed/damaged tissues.

Significant elevations in T_REG_ absolute counts and proportions were observed RACE + 1 vs. PRE-RACE. The function of these cells is primarily to regulate the immune response to infection and inflammation (Littringer et al. 2018; Lei et al. 2015), and the elevation of these cells in the peripheral blood 24 h post-race may indicate upregulated production of these cells to control inflammatory processes, or an active transport of these cells from lymph stores into the blood for re-direction to inflamed tissue (such as muscle and lungs). An alternate role for these cells in the context of recovery from extreme exercise, could be a contribution to repair and regeneration (Li et al. 2018). Recent evidence shows that these cells contain potent regenerative proteins, such as amphiregulin (Liu et al. 2022; Zaiss et al. 2015) which can promote tissue repair through epidermal growth factor signalling (Zaiss et al. 2015) and have been implicated in myocardial muscle repair post-myocardial infarction (Zhuang et al. 2022) as well as wound healing (Zaiss et al. 2019). Therefore, T_REG_ elevations within 24 h post-event could be contributing to a muscle tissue remodelling process, as well as suppressing macrophage- and other T-cell mediated inflammatory responses. Recently, Langston et al. (2023) demonstrated the role of T_REG_ in muscle post-exercise, with T_REG_ infiltration into skeletal muscle post-exercise promoting the long-term exercise training aerobic adaptations. However, this study was performed in mice, and thus human studies should now be undertaken to elucidate the role of T_REG_ changes with exercise in muscle adaptation. It must be noted that in this study, T_REG_ were measured as CD3+CD4+CD25+CD127−, and we did not include FoxP3 in our flow cytometry assay. CD127(−) was used to enumerate T_REG_ cells in our sample, as CD127 is downregulated in these cells and correlates well with T_REG_ suppressor functions (Liu et al. 2006; Yu et al. 2012), and CD4+CD25+CD127− cells were found to have greater suppressive function than broadly CD4+CD25+ T-cells (Yu et al. 2012). However, some CD127+ T-cells may also express FoxP3 (Klein et al. 2010), and therefore, the CD127low/− phenotype may be excluding a small proportion of T_REG_ cells in our study.

CD4+ and CD8+ EM absolute counts were reduced 48 h post-race, resulting in proportional increases in CD4+ NA cells. This drop in CD4+ and CD8+ EM absolute count could be explained by (1) selective apoptosis of these cells, or (2) egress of these cells into peripheral tissues at this timepoint. It is known that a small proportion of T-cells acutely express pro-apoptotic markers (Navalta et al. 2013; Kruger et al. 2016), with high intensity exercise preferentially promoting apoptosis in highly differentiated subsets, such as CD4+ and CD8+ EM and EMRA cells (Kruger et al. 2016). However, as we did not observe declines in CD4+ or CD8+ EMRA cells in this study, apoptosis may not be the only reason we observed changes in T-cells.

Upon exercise cessation, T-cells egress from the circulation into peripheral tissues (Kruger and Mooren 2007), with highly differentiated subsets displaying preferential egress (Graff et al. 2018), likely mediated by greater β_2_ adrenergic receptor expression on such subsets (Graff et al. 2018). Whilst we observed a reduction in CD4+ and CD8+ EM subsets 48 h post-exercise, it is likely that the reason for this reduction differs to that observed minutes post-exercise. The reduction of these cells in the circulation 48 h post-exercise is most likely due to trafficking to sites of muscle damage (Deyhle et al. 2020), with evidence suggesting an accumulation of CD4+, CD8+ T-cells with an effector phenotype in damaged skeletal muscle tissue in male Lewis rats (Deyhle et al. 2020), as well as in ultra-endurance athletes after a 24 h endurance bout of exercise (Marklund et al. 2013). Both studies documented elevations in skeletal muscle infiltration of CD8+ T-cells, with the former demonstrating a greater infiltration of CD4+ T-cells than CD8+ T-cells (Deyhle et al. 2020).

There is some argument for the reduction in CD4+ and CD8+ EM cells in the circulation to reflect suppressed immune function. However, this has been debated extensively (Campbell and Turner 2018; Simpson et al. 2020). The participants in the current study completed a 28-day upper respiratory tract infection (URTI) symptom questionnaire after the event (data not shown; Wisconsin Upper Respiratory Symptom Survey WURSS-11) (Obasi et al. 2014). Out of ten participants, 3 reported feeling sick within the 28 days, and this was unrelated to extent of changes within the T-cell phenotypes. This study was not designed to assess immune function and infection risk in these individuals, and thus more robust measures of URTI infections/symptoms should be incorporated into larger studies of this sort, as well as including appropriate controls. As a result, we cannot conclude whether the changes in EM absolute counts and proportions were resulting in elevated infection risk.

## Limitations

In this study, dietary behaviours post-race were not controlled, however, participants were encouraged to keep the same evening and morning routine for each blood sampling timepoint. Due to the event being a race in nature, intensity of the exercise (swim, cycle, run) could not be controlled, therefore the high variability in the T-cell data may be due to differences in finishing time and/or relative intensity. Inflammatory biomarkers, including markers of tissue damage, were not evaluated in this study, and therefore we can only speculate that the immunological response observed stems from tissue damage and inflammation. However, as exercise-induced inflammation and muscle damage is documented extensively elsewhere (Rubio-Arias et al. 2019; Turner et al. 2013; Marklund et al. 2013), we are confident these are related.

## Conclusion

A half ironman ultra-endurance event increased circulating T_REG_ populations and reduced circulating differentiated T-cells (EM subsets). These data reflect possible T-cell specific inflammatory processes, including trafficking of key cells to damaged and inflamed tissue, and immunoregulatory pathways, with T_REG_ subset elevations as a potential means to regulate inflammatory activity.

## Data Availability

Upon acceptance of this manuscript, data will be deposited open access and freely available via Heriot-Watt University.

## Abbreviations

ANOVA: Analysis of variance
BLa: Blood lactate
CM: Central memory T-cells
CRP: C-reactive protein
EM: Effector memory T-cells
HR: Heart rate
IM70.3: Half ironman triathlon (70.3 miles)
NA: Naïve T-cells
TEMRA: Terminally differentiated T-cells
T_REG_: Regulatory T-cells
PBMC: Peripheral blood mononuclear cells
URTI: Upper respiratory tract infection
$\dot{V}$O_2_max: Maximum oxygen uptake
